# Supplementary material for: Healthcare Workers’ Knowledge and Perception of the SARS-CoV-2 Omicron Variant: A Multinational Cross-Sectional Study
Source: Healthcare (Basel). 2022 Feb 25;10(3):438. doi: 10.3390/healthcare10030438 (PMC8951382; doi:10.3390/healthcare10030438)
Supplement: Supplementary file 1 [file healthcare-10-00438-s001.zip › Supplementary File S1.pdf]

Awareness, Knowledge and Perceptions regarding SARS-CoV-2 Omicron variant

Dear health professional,

The recent changes in the COVID-19 epidemic have led to the emergence of a new SARS-CoV-2 Variant of Concern (VOC), named "Omicron," and the number of Omicron cases were consistently increasing around the world. The health professional's knowledge is of utmost importance to prevent and control the spreading of the SARS-CoV-2 Omicron Variant of COVID-19. In this regard, we would likely to know the level of knowledge and Perceptions of health professionals about the SARS-CoV-2 Omicron Variant; this can provide a greater opportunity to understand the existing knowledge gaps about the novel Omicron variant and to scale up the interventional strategies. Thus, we invite you to participate in this short survey and provide your valuable opinion regarding the SARS-CoV-2 Omicron variant.

This survey contains 17 questions that are focused on investigating the various domains of knowledge and perception about the SARS-CoV-2 Omicron Variant. We request you to provide honest responses to the questions.

By clicking the below link, you agree to participate voluntarily in this survey and give your consent to use your anonymous data for research.

You may exit the survey at any stage and no compensation will be provided.

If you have any further queries, please write to me @xxxx

Thanking you in advance

Sincerely

XXXXX

Sociodemographic characteristics

Q1 What is your gender (D1)

○ Male (1)

○ Female (2)

Q2 What is your age (years) (D2)

○ 18 - 24 (2)

○ 25 - 34 (3)

○ 35 - 44 (4) X

○ 45 - 54 (5)

○ 55 - 64 (6)

○ 65 - 74 (7)

○ >74 (60)

Q3 Country (D3)

____

Q4 What is your profession (D4)

____

Q5 Have you heard of SARS-CoV-2 Omicron Variant (A1)

○ Yes (1) X

○ No (2)

Q6 Have you attended any lectures/discussions about the SARS-CoV-2 Omicron Variant? (A2)

○ Yes (1)

○ No (2) X

Q7 On a scale of 1 "least used sources" to 4 "most used sources", how do you rank your sources of information about the SARS-CoV-2 Omicron Variant as a global emergency?

	Least used (1)	Sometimes (2)	More often (3)	Most used (4)
News, Media (TV, Radio, newspapers etc.., (1)	○	○	○	○
Social media (Facebook, Twitter, Whatsapp, YouTube, Instagram, Snapchat…) (2)	○	○	○	○
Official government websites (MOH, WHO, CDC…..) (3)	○	○	○	○
Family member, colleague or friend (4)	○	○	○	○

Q8: Can you identify WHO designated SARS-CoV-2 Variant of Concern (VOCs) (K1)

**WHO designation**	**Country of Origin**
ALPHA (B.1.1.7)	United Kingdom (1)
BETA (B.1.351)	South Africa (2)
GAMMA (P.1)	Brazil (3)
DELTA (B.1.6172.2	India (4)
OMICRON (B.1.1.529)	South Africa (5)

Q9 The SARS-CoV-2 Omicron Variant was first reported to the WHO from (K2)

- India (1)
- China (2)
- United States of America (3)
- South Africa (4) X
- United Kingdom (5)
- None of the above (6)

Q10 Symptoms of SARS-COV-2 Omicron Variant are all except: (K3)

- Headache (1)
- Cough (2)
- Runny/stuffy nose (3)
- Sore throat (4)
- Skin rash (5) X

Q11 SARS-CoV-2 Omicron Variant is transmissible to: (K4)

- ○ Children (1)
- ○ Young adults (2)
- ○ Middle-aged adults (3)
- ○ Older people (4)
- ○ All the above (5) X

Q12 Which of the following measures can protect against the SARS-CoV-2 Omicron Variant: (K5)

- ○ Wear well-fitting masks (1)
- ○ Hand hygiene (2)
- ○ Physical distancing (3)
- ○ Improving ventilation of indoor spaces (4)
- ○ Avoiding crowded spaces (5)
- ○ COVID-19 vaccination (6)
- ○ All the above (7) X

Q13 Taking 2-doses of the COVID-19 vaccine is important? (K6)

- ○ Yes (1) X
- ○ No (2)

Q14 Do you believe the currently available COVID-19 vaccines offer protection against the SARS-CoV-2 Omicron Variant? (P1)

- ○ Yes (1) X
- ○ No (2)
- ○ Not sure (3)

Q15 Do you believe COVID-19 booster dose can offer protection against SARS-CoV-2 Omicron Variant? (P2)

○ Yes (1) X

○ No (2)

○ Not sure (3)

Q16 Do you think SARS-CoV-2 mutations could alter the response to vaccines, treatments, and transmissibility. (P3)

○ Yes (1) X

○ No (2)

○ Not sure (3)

Q17 Answer the following True or false questions (P4-P10)

	True	False
Both vaccinated, and unvaccinated people are vulnerable to the SARS-CoV-2 Omicron Variant (1)	○ X	○
Travel bans can limit the global spread of SARS-CoV-2 Omicron Variant (2)	○	○ X
Older people or those with comorbidities should be advised to postpone travel (3)	○ X	○
Steroids are effective against severe SARS-CoV-2 Omicron variant (4)	○	○ X
A COVID-19 rapid antigen test is reliable to detect the SARS-CoV-2 Omicron Variant. (5)	○	○ X
Masks offer protection against all SARS-CoV-2 variants (6)	○ X	○
Countries should accelerate the COVID-19 vaccination program (7)	○ X	○
