# Supplementary figures and images for: Healthcare Workers’ Knowledge and Perception of the SARS-CoV-2 Omicron Variant: A Multinational Cross-Sectional Study
Source: Healthcare (Basel). 2022 Feb 25;10(3):438. doi: 10.3390/healthcare10030438 (PMC8951382; doi:10.3390/healthcare10030438)

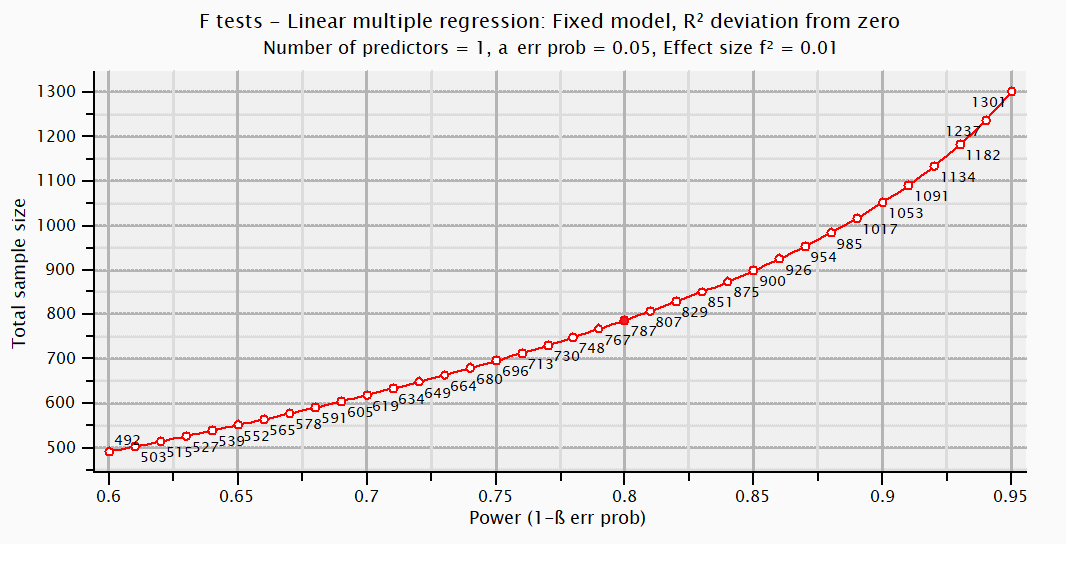

Supplement: Supplementary file 1 [file healthcare-10-00438-s001.zip › Supplementary Figure S1.png]
